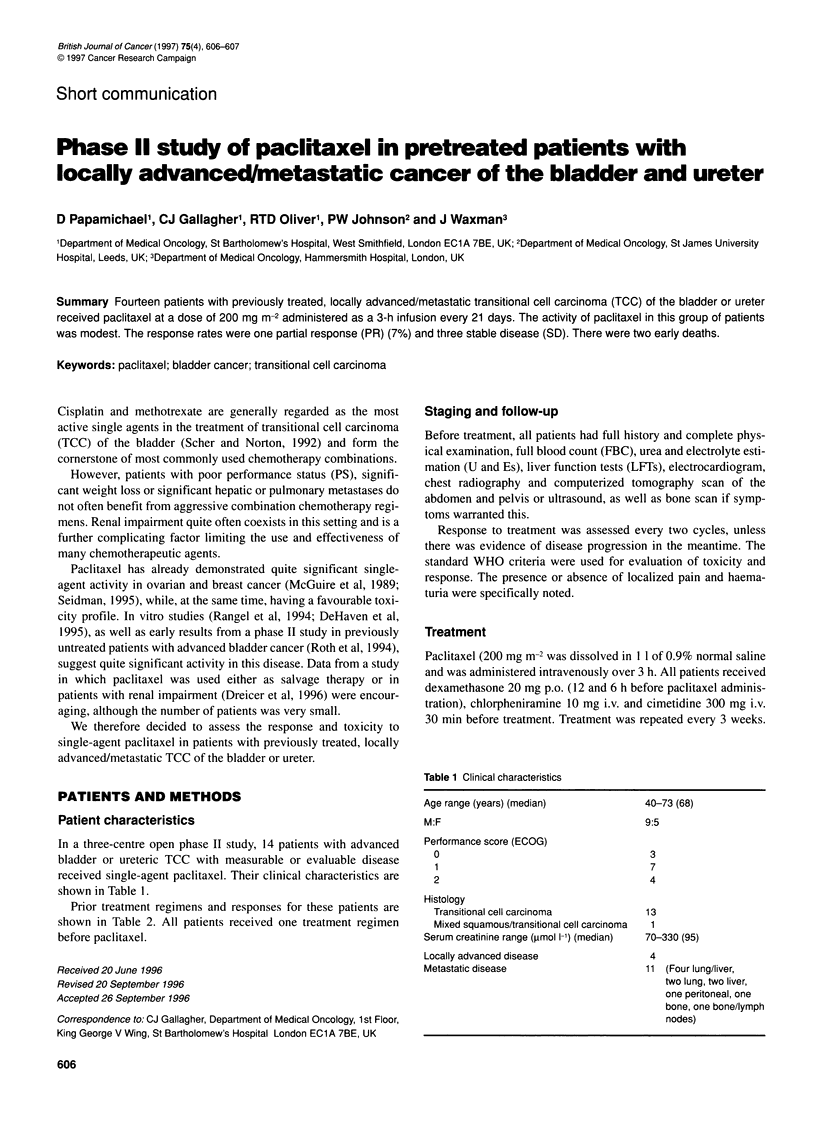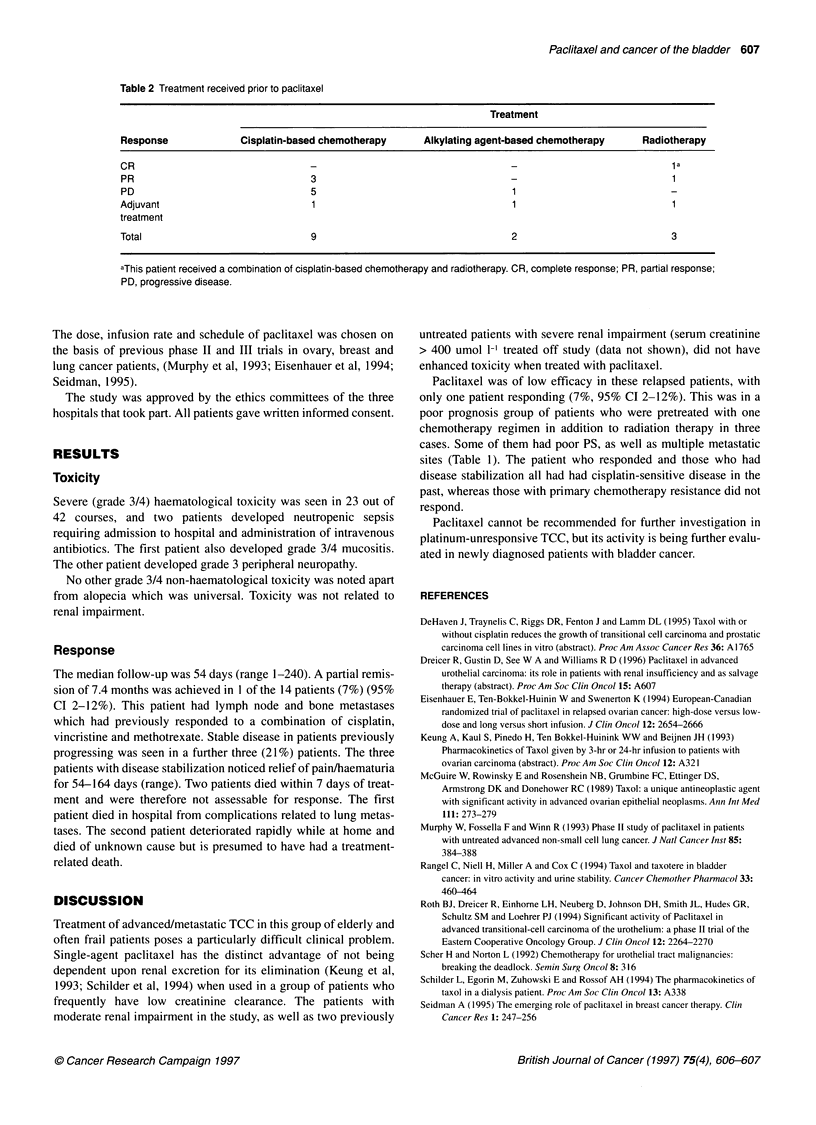# Phase II study of paclitaxel in pretreated patients with locally advanced/metastatic cancer of the bladder and ureter.

**DOI:** 10.1038/bjc.1997.106

**Published:** 1997

**Authors:** D. Papamichael, C. J. Gallagher, R. T. Oliver, P. W. Johnson, J. Waxman

**Affiliations:** Department of Medical Oncology, St Bartholomew's Hospital, West Smithfield, London, UK.

## Abstract

Fourteen patients with previously treated, locally advanced/metastatic transitional cell carcinoma (TCC) of the bladder or ureter received paclitaxel at a dose of 200 mg m-2 administered as a 3-h infusion every 21 days. The activity of paclitaxel in this group of patients was modest. The response rates were one partial response (PR) (7%) and three stable disease (SD). There were two early deaths.


British Journal of Cancer (1997) 75(4), 606–607
© 1997 Cancer Research Campaign

Short communication

### Phase II study of paclitaxel in pretreated patients with locally advanced/metastatic cancer of the bladder and ureter

**D Papamichael[1], CJ Gallagher[1], RTD Oliver[1], PW Johnson[2] and J Waxman[3]**

[1]Department of Medical Oncology, St Bartholomew's Hospital, West Smithfield, London EC1A 7BE, UK; [2]Department of Medical Oncology, St James University Hospital, Leeds, UK; [3]Department of Medical Oncology, Hammersmith Hospital, London, UK

**Summary** Fourteen patients with previously treated, locally advanced/metastatic transitional cell carcinoma (TCC) of the bladder or ureter received paclitaxel at a dose of 200 mg $m^{-2}$ administered as a 3-h infusion every 21 days. The activity of paclitaxel in this group of patients was modest. The response rates were one partial response (PR) (7%) and three stable disease (SD). There were two early deaths.

**Keywords:** paclitaxel; bladder cancer; transitional cell carcinoma

Cisplatin and methotrexate are generally regarded as the most active single agents in the treatment of transitional cell carcinoma (TCC) of the bladder (Scher and Norton, 1992) and form the cornerstone of most commonly used chemotherapy combinations.

However, patients with poor performance status (PS), significant weight loss or significant hepatic or pulmonary metastases do not often benefit from aggressive combination chemotherapy regimens. Renal impairment quite often coexists in this setting and is a further complicating factor limiting the use and effectiveness of many chemotherapeutic agents.

Paclitaxel has already demonstrated quite significant single-agent activity in ovarian and breast cancer (McGuire et al, 1989; Seidman, 1995), while, at the same time, having a favourable toxicity profile. In vitro studies (Rangel et al, 1994; DeHaven et al, 1995), as well as early results from a phase II study in previously untreated patients with advanced bladder cancer (Roth et al, 1994), suggest quite significant activity in this disease. Data from a study in which paclitaxel was used either as salvage therapy or in patients with renal impairment (Dreicer et al, 1996) were encouraging, although the number of patients was very small.

We therefore decided to assess the response and toxicity to single-agent paclitaxel in patients with previously treated, locally advanced/metastatic TCC of the bladder or ureter.

### PATIENTS AND METHODS

### Patient characteristics

In a three-centre open phase II study, 14 patients with advanced bladder or ureteric TCC with measurable or evaluable disease received single-agent paclitaxel. Their clinical characteristics are shown in Table 1.

Prior treatment regimens and responses for these patients are shown in Table 2. All patients received one treatment regimen before paclitaxel.

*Received 20 June 1996*
*Revised 20 September 1996*
*Accepted 26 September 1996*

*Correspondence to:* CJ Gallagher, Department of Medical Oncology, 1st Floor, King George V Wing, St Bartholomew's Hospital London EC1A 7BE, UK

### Staging and follow-up

Before treatment, all patients had full history and complete physical examination, full blood count (FBC), urea and electrolyte estimation (U and Es), liver function tests (LFTs), electrocardiogram, chest radiography and computerized tomography scan of the abdomen and pelvis or ultrasound, as well as bone scan if symptoms warranted this.

Response to treatment was assessed every two cycles, unless there was evidence of disease progression in the meantime. The standard WHO criteria were used for evaluation of toxicity and response. The presence or absence of localized pain and haematuria were specifically noted.

### Treatment

Paclitaxel (200 mg $m^{-2}$ was dissolved in 1 l of 0.9% normal saline and was administered intravenously over 3 h. All patients received dexamethasone 20 mg p.o. (12 and 6 h before paclitaxel administration), chlorpheniramine 10 mg i.v. and cimetidine 300 mg i.v. 30 min before treatment. Treatment was repeated every 3 weeks.

**Table 1** Clinical characteristics

| | |
|---|---|
| Age range (years) (median) | 40–73 (68) |
| M:F | 9:5 |
| Performance score (ECOG) | |
| 0 | 3 |
| 1 | 7 |
| 2 | 4 |
| Histology | |
| Transitional cell carcinoma | 13 |
| Mixed squamous/transitional cell carcinoma | 1 |
| Serum creatinine range (μmol $l^{-1}$) (median) | 70–330 (95) |
| Locally advanced disease | 4 |
| Metastatic disease | 11 (Four lung/liver, two lung, two liver, one peritoneal, one bone, one bone/lymph nodes) |

**Table 2** Treatment received prior to paclitaxel

| | Treatment | | |
|---|---|---|---|
| Response | Cisplatin-based chemotherapy | Alkylating agent-based chemotherapy | Radiotherapy |
| CR | – | – | 1[a] |
| PR | 3 | – | 1 |
| PD | 5 | 1 | – |
| Adjuvant treatment | 1 | 1 | 1 |
| Total | 9 | 2 | 3 |

[a]This patient received a combination of cisplatin-based chemotherapy and radiotherapy. CR, complete response; PR, partial response; PD, progressive disease.

The dose, infusion rate and schedule of paclitaxel was chosen on the basis of previous phase II and III trials in ovary, breast and lung cancer patients, (Murphy et al, 1993; Eisenhauer et al, 1994; Seidman, 1995).

The study was approved by the ethics committees of the three hospitals that took part. All patients gave written informed consent.

### RESULTS

### Toxicity

Severe (grade 3/4) haematological toxicity was seen in 23 out of 42 courses, and two patients developed neutropenic sepsis requiring admission to hospital and administration of intravenous antibiotics. The first patient also developed grade 3/4 mucositis. The other patient developed grade 3 peripheral neuropathy.

No other grade 3/4 non-haematological toxicity was noted apart from alopecia which was universal. Toxicity was not related to renal impairment.

### Response

The median follow-up was 54 days (range 1–240). A partial remission of 7.4 months was achieved in 1 of the 14 patients (7%) (95% CI 2–12%). This patient had lymph node and bone metastases which had previously responded to a combination of cisplatin, vincristine and methotrexate. Stable disease in patients previously progressing was seen in a further three (21%) patients. The three patients with disease stabilization noticed relief of pain/haematuria for 54–164 days (range). Two patients died within 7 days of treatment and were therefore not assessable for response. The first patient died in hospital from complications related to lung metastases. The second patient deteriorated rapidly while at home and died of unknown cause but is presumed to have had a treatment-related death.

### DISCUSSION

Treatment of advanced/metastatic TCC in this group of elderly and often frail patients poses a particularly difficult clinical problem. Single-agent paclitaxel has the distinct advantage of not being dependent upon renal excretion for its elimination (Keung et al, 1993; Schilder et al, 1994) when used in a group of patients who frequently have low creatinine clearance. The patients with moderate renal impairment in the study, as well as two previously untreated patients with severe renal impairment (serum creatinine > 400 umol $l^{-1}$ treated off study (data not shown), did not have enhanced toxicity when treated with paclitaxel.

Paclitaxel was of low efficacy in these relapsed patients, with only one patient responding (7%, 95% CI 2–12%). This was in a poor prognosis group of patients who were pretreated with one chemotherapy regimen in addition to radiation therapy in three cases. Some of them had poor PS, as well as multiple metastatic sites (Table 1). The patient who responded and those who had disease stabilization all had had cisplatin-sensitive disease in the past, whereas those with primary chemotherapy resistance did not respond.

Paclitaxel cannot be recommended for further investigation in platinum-unresponsive TCC, but its activity is being further evaluated in newly diagnosed patients with bladder cancer.

### REFERENCES

DeHaven J, Traynelis C, Riggs DR, Fenton J and Lamm DL (1995) Taxol with or without cisplatin reduces the growth of transitional cell carcinoma and prostatic carcinoma cell lines in vitro (abstract). *Proc Am Assoc Cancer Res* **36:** A1765

Dreicer R, Gustin D, See W A and Williams R D (1996) Paclitaxel in advanced urothelial carcinoma: its role in patients with renal insufficiency and as salvage therapy (abstract). *Proc Am Soc Clin Oncol* **15:** A607

Eisenhauer E, Ten-Bokkel-Huinin W and Swenerton K (1994) European-Canadian randomized trial of paclitaxel in relapsed ovarian cancer: high-dose versus low-dose and long versus short infusion. *J Clin Oncol* **12:** 2654–2666

Keung A, Kaul S, Pinedo H, Ten Bokkel-Huinink WW and Beijnen JH (1993) Pharmacokinetics of Taxol given by 3-hr or 24-hr infusion to patients with ovarian carcinoma (abstract). *Proc Am Soc Clin Oncol* **12:** A321

McGuire W, Rowinsky E and Rosenshein NB, Grumbine FC, Ettinger DS, Armstrong DK and Donehower RC (1989) Taxol: a unique antineoplastic agent with significant activity in advanced ovarian epithelial neoplasms. *Ann Int Med* **111:** 273–279

Murphy W, Fossella F and Winn R (1993) Phase II study of paclitaxel in patients with untreated advanced non-small cell lung cancer. *J Natl Cancer Inst* **85:** 384–388

Rangel C, Niell H, Miller A and Cox C (1994) Taxol and taxotere in bladder cancer: in vitro activity and urine stability. *Cancer Chemother Pharmacol* **33:** 460–464

Roth BJ, Dreicer R, Einhorne LH, Neuberg D, Johnson DH, Smith JL, Hudes GR, Schultz SM and Loehrer PJ (1994) Significant activity of Paclitaxel in advanced transitional-cell carcinoma of the urothelium: a phase II trial of the Eastern Cooperative Oncology Group. *J Clin Oncol* **12:** 2264–2270

Scher H and Norton L (1992) Chemotherapy for urothelial tract malignancies: breaking the deadlock. *Semin Surg Oncol* **8:** 316

Schilder L, Egorin M, Zuhowski E and Rossof AH (1994) The pharmacokinetics of taxol in a dialysis patient. *Proc Am Soc Clin Oncol* **13:** A338

Seidman A (1995) The emerging role of paclitaxel in breast cancer therapy. *Clin Cancer Res* **1:** 247–256

© Cancer Research Campaign 1997